# From the identification of actionable molecular targets to the generation of faithful neuroblastoma patient-derived preclinical models

**DOI:** 10.1186/s12967-024-04954-w

**Published:** 2024-02-13

**Authors:** Mario Capasso, Chiara Brignole, Vito A. Lasorsa, Veronica Bensa, Sueva Cantalupo, Enrico Sebastiani, Alessandro Quattrone, Eleonora Ciampi, Marianna Avitabile, Angela R. Sementa, Katia Mazzocco, Barbara Cafferata, Gabriele Gaggero, Valerio G. Vellone, Michele Cilli, Enzo Calarco, Elena Giusto, Patrizia Perri, Sanja Aveic, Doriana Fruci, Annalisa Tondo, Roberto Luksch, Rossella Mura, Marco Rabusin, Francesco De Leonardis, Monica Cellini, Paola Coccia, Achille Iolascon, Maria V. Corrias, Massimo Conte, Alberto Garaventa, Loredana Amoroso, Mirco Ponzoni, Fabio Pastorino

**Affiliations:** 1 Department of Medical Biotechnology, University of Naples Federico II, 80138 Naples, Italy; 2 Laboratory of Experimental Therapies in Oncology, IRCCS Istituto Giannina Gaslini, Via G. Gaslini 5, 16147 Genoa, Italy; 3 CEINGE Advanced Biotecnology, 80138 Naples, Italy; 4 CIBIO, University of Trento, 38122 Trento, Italy; 5 Pathological Anatomy, IRCCS Istituto Giannina Gaslini, 16147 Genoa, Italy; 6 Animal Facility, IRCCS Policlinico San Martino, 16100 Genoa, Italy; 7 Pediatric Research Institute Città Della Speranza, 35127 Padua, Italy; 8 Department of Emato-Oncology, Bambino Gesù Children’s Hospital, 00146 -Rome, Italy; 9 Department of Emato-Oncology, Anna Meyer Children’s Hospital, 50139 Florence, Italy; 10 Emato-Oncology Unit, Fondazione IRCCS Istituto Nazionale Dei Tumori, 20133 Milan, Italy; 11 Emato-Oncology Unit, Azienda Ospedaliera Brotzu, 09047 Cagliari, Italy; 12 Pediatric Department, Institute for Maternal and Child Health, IRCCS Burlo Garofolo, 34137 Trieste, Italy; 13 Department of Pediatric Oncology, Polyclinic Hospital, 70124 Bari, Italy; 14 Emato-Oncology Unit, University-Hospital Polyclinic of Modena, 41124 Modena, Italy; 15 University-Hospital of Marche, Presidio Ospedaliero “G. Salesi”, 60126 Ancona, Italy; 16 Clinical Oncology Unit, IRCCS Istituto Giannina Gaslini, 16147 -Genoa, Italy

**Keywords:** Precision medicine, Neuroblastoma, Target therapy, Preclinical models, next generation sequencing

## Abstract

**Background:**

Neuroblastoma (NB) represents the most frequent and aggressive form of extracranial solid tumor of infants. Although the overall survival of patients with NB has improved in the last years, more than 50% of high-risk patients still undergo a relapse. Thus, in the era of precision/personalized medicine, the need for high-risk NB patient-specific therapies is urgent.

**Methods:**

Within the PeRsonalizEd Medicine (PREME) program, patient-derived NB tumors and bone marrow (BM)-infiltrating NB cells, derived from either iliac crests or tumor bone lesions, underwent to histological and to flow cytometry immunophenotyping, respectively. BM samples containing a NB cells infiltration from 1 to 50 percent, underwent to a subsequent NB cells enrichment using immune-magnetic manipulation. Then, NB samples were used for the identification of actionable targets and for the generation of 3D/tumor-spheres and Patient-Derived Xenografts (PDX) and Cell PDX (CPDX) preclinical models.

**Results:**

Eighty-four percent of NB-patients showed potentially therapeutically targetable somatic alterations (including point mutations, copy number variations and mRNA over-expression). Sixty-six percent of samples showed alterations, graded as “very high priority”, that are validated to be directly targetable by an approved drug or an investigational agent. A molecular targeted therapy was applied for four patients, while a genetic counseling was suggested to two patients having one pathogenic germline variant in known cancer predisposition genes. Out of eleven samples implanted in mice, five gave rise to (C)PDX, all preserved in a local PDX Bio-bank. Interestingly, comparing all molecular alterations and histological and immunophenotypic features among the original patient’s tumors and PDX/CPDX up to second generation, a high grade of similarity was observed. Notably, also 3D models conserved immunophenotypic features and molecular alterations of the original tumors.

**Conclusions:**

PREME confirms the possibility of identifying targetable genomic alterations in NB, indeed, a molecular targeted therapy was applied to four NB patients. PREME paves the way to the creation of clinically relevant repositories of faithful patient-derived (C)PDX and 3D models, on which testing precision, NB standard-of-care and experimental medicines.

**Supplementary Information:**

The online version contains supplementary material available at 10.1186/s12967-024-04954-w.

## Introduction

Neuroblastoma (NB) represents the most frequent form of extra-cranial solid tumour of infants, responsible for 15% of childhood cancer deaths [1]. In NB, recurrent large genomic alterations such as *MYCN* amplification, 1p and 11q deletions, unbalanced 17q gain [2], *TERT* rearrangements [3] and 19p loss [4] are reliable predictors of poor clinical outcome. Somatic point mutations and large genomic aberrations of *ALK *[5] and *ATRX *[2] occur relatively frequent in NB and are considered promising therapeutic targets. Regulatory noncoding variants have been reported as drivers of NB with the potential to predict patient prognosis [6, 7], while gene expression signatures are shown to be markers of clinical outcome in high-risk patients [8, 9]. All these genomic findings have contributed to better stratify patients according to their disease risk and to indicate the more effective treatment. In addition, large genetic association studies have unraveled potentially clinically actionable rare and common germline variants [10], such as NB predisposing loss of function mutations in genes belonging to homologous recombination pathway [11]. Indeed, overall survival of children with cancer has dramatically improved over the past years but the survival rates of refractory and/or relapsing high-risk NB patients remain obstinately low [1]. For this reason, achieving effective NB treatment represents one of the major challenges in pediatric oncology.

In the era of precision/personalized medicine, the need for patient-specific therapies is crucial. Recently, diverse prospective precision medicine programs [12–17] by applying next generation sequencing (NGS) approaches, on large cohorts of pediatric patients, have revealed a rate of actionable molecular alterations that justify the development of predictive biomarker-driven trials for childhood cancer.

PREME (PeRsonalizEdMEdicine) is an Italian, multicentric, prospective and non-profit project composed of sub-projects focused on the design of innovative therapeutic strategies for patients with NB. PREME is developed by professionals with complementary competences in preclinical research, genomic characterization and care of patients with NB, different skills in the biological, veterinary, genomic, bioinformatics and clinical fields. This synergic cooperation creates the opportunity to carry out prospective clinical evaluations with a propensity to (i) identify new biomarkers and monitor treatment response, (ii) study oncogenes in pre-clinical pediatric in vivo tumor models, (iii) select the most appropriate pharmacological combinations, (iv) develop new bioinformatics approaches to investigate tumor evolution, and finally, (v) design the tools for patient-tailored precision medicine. The ultimate goal of PREME is to identify new druggable molecular targets by using the preclinical platforms here proposed for focused therapeutic applications.

Here, we report the first Italian results consisting of whole exome, panel genes and transcriptome analysis in patient-derived NB tumors and bone marrow (BM)-infiltrating NB cells, derived from either iliac crests or tumor bone lesions, to identify relevant somatic and germline aberrations in NB patients to inform treatment. We also show the results of genomic, histological and immunophenotypic characterization of tumor patient-derived xenograft (PDX) and 3D tumor-sphere models that are essential to systematically prioritize and rationally assess novel anti-tumor agents.

## Methods

### Study design and workflow

**Fig. 1:**
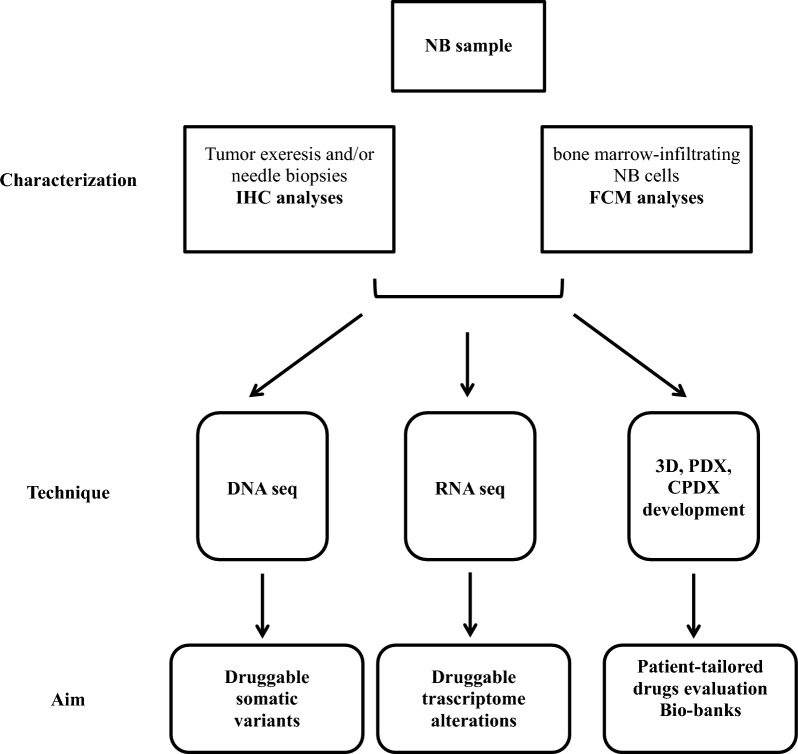
Schematic representation of PREME. *NB* neuroblastoma, *IHC* immunohistochemistry, *FCM* flow cytometry, *PDX* Patient-Derived Xenograft, *CDPX* Cell Patient-Derived Xenograft, *3D* patient-derived tumor-spheres

**Fig. 2:**
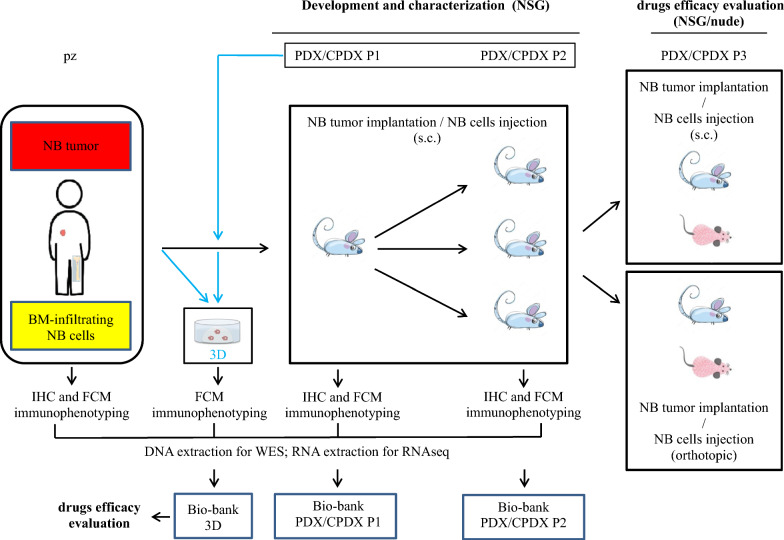
Development, characterization, and therapeutic use of PDX/CPDX and 3D models. *IHC* immunohistochemistry, *FCM* flow cytometry, *PDX* Patient-Derived Xenograft, *CDPX* Cell Patient-Derived Xenograft, *3D* patient-derived tumor-spheres, *NB* neuroblastoma, *BM* Bone Marrow derived from either iliac crests or tumor bone lesions, *NSG* NOD/SCID/IL2Rgammanull mice, nude athymic nude/nude mice, s.c. subcutaneous implant/injection of NB tumor fragments or NB cell suspension derived from tumor-infiltrated BM, orthotopic implantation/injection of NB tumor fragments or NB cell suspension derived from P2 generation mice, in the adrenal gland, *WES* whole exome sequencing

In the first part of activity within the PREME program, tumors and bone marrow (BM)-infiltrating cells derived from 18 NB patients were obtained from different Associazione Italiana Ematologia Oncologia Pediatrica (AIEOP) centers. A schematic representation of PREME actions and aims is reported in Figs. 1 and 2.

The samples, and the corresponding peripheral blood, derived from tumor masses of patients with relapsed NB (n = 13) and from BM-infiltrating NB cells of patients at onset or at relapse (n = 1 and n = 4, respectively) (Table 1). For one patient (Pz#2), we analyzed DNA of an additional consecutive relapsed tumor (Pz#2bis).

**Table 1:** Characteristics and genomic applications of tumors and their respective derived preclinical models

Pz code	Gender	Age at diagnosis (months)	Histopathologic classification of the tumor	INRG-SS Stage	Immunohistochemistry						Flow Cytometry					WES-g	WES-t	CGP	RNAseq
					*MYCN*	CD45	CD56	TH	Phox-2B	S100	CD45	CD56	GD2	B7-H3	NCL	yes (y)	yes (y)	yes (y)	yes (y)
pz#0	M	35	NB, Schwannian-stroma poor, poorly differentiated	M	No ampl	+	+	+	+	−	NP	NP	NP	NP	NP	y	y		
pz#1	M	41	NB, Schwannian-stroma poor, poorly differentiated	M	NP	+	+	+	+	NP	NP	NP	NP	NP	NP		y		
Pz#2	F	8	NB, Schwannian-stroma poor, poorly differentiated	L2	No ampl	+	+	+	+	NP	NP	NP	NP	NP	NP	y	y		y
**Pz#2bis**					No ampl	NP	NP	NP	NP	NP	−	+	NP	+	−		y		
Pz#3	M	46	NB, Schwannian-stroma poor, undifferentiated	M	Ampl	+	+	+	+	+	+	+	+	+	+	y	y		y
PDX#3-P1			NB, Schwannian-stroma poor, undifferentiated		Ampl	−	+	+	+	−	−	+	+	+	+		y		y
PDX#3-P2			NB, Schwannian-stroma poor, undifferentiated		Ampl	−	+	+	+	−	−	+	+	+	+		y		y
PDX#3-P2-3D					NP	NP	NP	NP	NP	NP	−	+	+	+	NP		y		
Pz#4	F	198	NB, Schwannian-stroma poor, undifferentiated	M	No ampl	+	+	+	−	NP	+	+	+	+	+	y	y		y
PDX#4-P1			NB, Schwannian-stroma poor, undifferentiated		No ampl	−	+	+	−	−	−	+	+	+	+		y		y
PDX#4-P2			NB, Schwannian-stroma poor, undifferentiated		No ampl	−	+	+	−	−	−	+	+	+	+		y		y
PDX#4-P2-3D					NP	NP	NP	NP	NP	NP	NP	NP	NP	NP	NP		y		
Pz#5	M	49	NB, Schwannian-stroma poor, undifferentiated	M	No ampl GNE	+	NP	+	+	NP	NP	NP	NP	NP	NP	y	y		
PDX#5-P1			NB, Schwannian-stroma poor, undifferentiated		No ampl GNE	−	+	+	+	−	NP	NP	NP	NP	NP		y		
PDX#5-P2			NB, Schwannian-stroma poor, poorly differentiated		gain	−	+	+	+	−	−	+	+	+	+		y		
PDX#5-P2-3D					NP	NP	NP	NP	NP	NP	NP	NP	NP	NP	NP				
Pz#6	M	45	NE	L2	No ampl	NP	NP	NP	NP	NP	NP	NP	NP	NP	NP	y	y		
Pz#7	M	172	NB, Schwannian-stroma poor, undifferentiated	L1	No ampl	+	+	+	±	−	NP	NP	NP	NP	NP	y	y		y
Pz#8	F	89	NB, Schwannian-stroma poor, undifferentiated	L2	No ampl	±	+	+	NE	−	+	+	+	+	+	y	y		y
PDX#8-P1			NB, Schwannian-stroma poor, undifferentiated		No ampl	−	+	+	+	−	−	+	+	+	+		y		y
PDX#8-P2			NB, Schwannian-stroma poor, undifferentiated		No ampl	−	+	+	+	−	−	+	+	+	+		y		
PDX#8-P2-3D					NP	NP	NP	NP	NP	NP	NP	NP	NP	NP	NP		y		
Pz#9	M	65	NB, Schwannian-stroma poor, undifferentiated	M	No ampl	+	+	+	−	−	NP	NP	NP	NP	NP	y	y		
Pz#10	F	51	NB, Schwannian-stroma poor, poorly differentiated	M	No ampl	+	+	+	+	−	NP	NP	NP	NP	NP	^y (CGP)		y	
Pz#11	F	1	NB, Schwannian-stroma poor, poorly differentiated	L1	No ampl	+	+	+	±	−	NP	NP	NP	NP	NP	y	y		y
Pz#14	F	101	NB, Schwannian-stroma poor, poorly differentiated	L1	No ampl	+	+	±	+	−	NP	NP	NP	NP	NP	y	y		y
**Pz#C**	F	22		M	Ampl	NP	NP	NP	NP	NP	+	+	+	+	+	y	y		y
Pz#C-3D					NP	NP	NP	NP	NP	NP	NP	NP	NP	NP	NP				
CPDX#C-P1			NB, Schwannian-stroma poor, undifferentiated		Ampl	−	+	+	+	−	−	+	+	+	±		y		y
CPDX#C-P1-3D					NP	NP	NP	NP	NP	NP	−	+	+	+	+				
CPDX#C-P2			NB, Schwannian-stroma poor, undifferentiated		Ampl	−	+	+	+	−	−	+	+	+	±		y		
CPDX#C-P2-3D					NP	NP	NP	NP	NP	NP	−	+	+	+	+		y		
**Pz#D**	M	68		M	NP	NP	NP	NP	NP	NP	+	+	+	+	+	y	y		
**Pz#F**	F	39		M	No ampl	NP	NP	NP	NP	NP	+	+	+	+	±	y		y	
**Pz#H**	M	39		M	Gain	NP	NP	NP	NP	NP	+	+	+	+	±	y		y	y
**Pz#I**	F	27		M	Ampl	NP	NP	NP	NP	NP	+	+	+	+	±	y		y	

All tumors derived from patients at relapsed except for Pz#C

In bold bone marrow-infiltrating NB cells

*NB* neuroblastoma, *Pz* patient, *PDX* Patient-Derived Xenografts, *CPDX* Cell Patient-Derived Xenografts, *3D* tumor-spheres, *M* male, *F* female, *ampl* amplified, *no ampl* not amplified, *GNE* gain not evaluable, *NP* not performed, *NE* not evaluable

+  positive, − negative, ±  slightly positive, *WES-g* whole exome sequencing of germline DNA, *WES-t* whole exome sequencing of tumor DNA, *CGP* cancer gene panel

^CPG of germline DNA has been performed for the Pz#10

Firstly, NB tumor samples evaluation and characterization was performed by histological (IHC) immunophenotyping (selection panel: CD45, CD56, TH, PHOX-2B, S100), while BM-infiltrating NB cells underwent to flow cytometry (FCM) immunophenotyping (selection panel: CD45, CD56, GD2, B7-H3, NCL). In both cases, *MYCN* status was evaluated by FISH. Then, based on the quantity and quality of the sample, as shown in Fig. 1, the biological material was divided following the reported order of priority:

(1) DNA extraction for subsequent DNAseq by Whole Exome Sequencing (WES) or Cancer Genes Panel sequencing (CGP), when the percentage of the neoplastic counterpart within the sample was over or below 60%, respectively;

(2) RNA extraction for subsequent RNAseq;

(3) Development of primary NB cell cultures (3D/patient-derived tumor-spheres) and of Patient-Derived and Cell Patient-Derived Xenograft (PDX and CPDX, respectively) models in mice;

(4) Creation of 3D and PDX/CPDX repositories.

### NB tumor characterization

#### Immunohistochemistry (IHC)

Tumor samples derived from 13 high-risk NB tumors at relapse (Table 1) underwent to paraffin embedding for IHC immunophenotyping. Tumor tissues Sects. (2 μm) were, indeed, de-paraffinized and incubated with the immune-cell marker CD45 (LCA) (Mouse Monoclonal Antibody (mAb)—Cell Marque/Roche), and with the NB markers CD56 (MRQ-42) (Rabbit mAb—Cell Marque/Roche), TH (F-11) (Mouse mAb—Santa Cruz Biotechnology), PHOX-2B (EPR14423) (rabbit mAb – Abcam) and S100 (4C4.9) (Mouse Monoclonal Antibody—Ventana / Roche). In some cases, primary Abs were diluted with Ab diluent Ventana / Roche (for TH mAb) or with BOND Primary Antibody Diluent—Leica (for PHOX-2B mAb). The ultraView Universal DAB detection kit from Ventana and the BOND Polimer Refine Detection kit from Leica (for PHOX-2B mAb) were used to detect the binding of primary antibodies. Sections were counterstained with HEMATOXYLIN -Ventana / Roche.

#### Flow cytometry (FCM)

Five BM samples, from NB patients at onset and relapse (Table 1), were subjected to immunophenotyping. Briefly, 60 µL of whole BM blood were dispensed in each tube and stained with the following monoclonal antibody (mAbs) panel: a-CD45 PE-Cy7 (mouse IgG1, clone 2D1; Invitrogen), a-CD56 APC (mouse IgG1, clone CMSSB; Invitrogen), a-GD2 PE (mouse IgG2a, clone 14G2a; Biolegend), a-B7-H3 PE (mouse IgG1, clone DCN.70; Biolegend), a-NCL AlexaFluor 488 (mouse IgG1, clone 364–5; Abcam). Samples were incubated for 25 min at 4 °C. Then, red cells were lysed using 1 × BD Pharm Lyse™ lysing solution (BD Biosciences, Franklin Lakes, New Jersey, USA) according to the manufacturer’s instructions. After a final washing step with PBS (1% FBS, 2 mM EDTA), samples were analyzed by FCM. The percentage of NB cells infiltrating the BM was defined as CD45^neg^/CD56^pos^/either GD2^pos^ or/and B7-H3^pos^.

The same procedure was followed for tumor samples when material was available. For this purpose, tumor mass was mechanically reduced to single cell suspension through the BD Medimachine System, by using sterile medicon 50 µm in size (BD Biosciences), and then filtered through a 70 µm cell strainer, and in following stained, as already described.

#### NB cells enrichment

BM samples containing a NB cells infiltration from 1 to 50 percent, underwent to a subsequent NB cells enrichment using immune-magnetic manipulation for a positive selection of GD2^pos^ or B7-H3^pos^ population of cells. Specifically, NB cells infiltrating the BM samples were incubated with human FcR blocking reagent (Miltenyi Biotec, Srl, Bologna, Italy), 10 min at 4 °C, to increase the specificity of immunofluorescent staining, and then labeled with either anti-GD2 PE or anti-B7-H3 PE mAbs. The decision of using either anti-GD2 or anti-B7-H3 mAb was based on the best expression of the respective antigens, as revealed by immunophenotyping. After being stained, cells were incubated with anti-mouse IgG microbeads, according to the manufacturer’s instruction (Miltenyi Biotec). MS or LS MACS separation columns (Miltenyi Biotec) were used to collect the fraction of labeled NB cells, as previously described [18, 19]. At the end of this procedure, the GD2^pos^ or B7-H3^pos^ fraction of NB cells was further analyzed to determine the percentage of CD45^pos^ cells contaminating the samples. After NB cells enrichment, at least 8 × 10^5^ NB cells were used for DNA/RNA extraction.

### Development of clinically relevant in vivo and* ex-vivo* preclinical models: PDX and 3D tumor-spheres generation

PDX were developed by implanting in male mice either fresh or thawed tumor fragments derived from NB tumors. For the first time to our knowledge, a PDX was also developed from fresh BM-infiltrating NB cells (Table 1, Figs. 1 and 2), and is reported, hereafter, as CPDX. 3D tumor-spheres were developed by using both cells derived from BM-infiltrating NB cells and cells derived from PDX/CPDX generations.

#### PDX and CPDX

After NB cells evaluation and characterization, followed by DNA and RNA extraction, the remaining sample, if available, was implanted or inoculated into NOD/SCID/IL2Rgamma^null^ (NSG) mice (Fig. 2). Specifically, NB tumor fragments derived from 10 patients at relapse (Pz#1, #2, #3, #4, #5, #7, #8, #9, #11 and #14), and NB cell suspension (3 × 10^6^ CD56^pos^ cells in FBS-free RPMI-1640 medium) derived from 1 patient with tumor-infiltrated BM at onset (Pz#C), were subcutaneously implanted or injected, respectively, in the left flank of NSG mice. Corning Matrigel matrix (Merck Life Science S.r.l. Milan, Italy) was used as support. Once the transplanted tumor had reached a diameter of 1–2 cm, it was removed. At any generation, a fragment of the raised PDX and CPDX tumor was phenotypically evaluated. Specifically, samples were both paraffin embedded for IHC evaluation, and mechanically reduced to single cell suspension for FCM evaluation, as reported earlier under FCM protocol. Moreover, samples were also genetically characterized (DNAseq and RNAseq) to determine any possible divergence from the original pattern of patient’s tumor. *MYCN* status was also evaluated by FISH. Once demonstrated the conserved tumor characteristics, another tumor fragment was frozen in 90% FBS/10% DMSO freezing solution to create a repository of PDX and CPDX with different genetic features, and to be used for drug screening and evaluation (Fig. 2).

#### 3D from BM-infiltrating NB cells and PDX/CPDX generations

After NB cells evaluation and characterization, followed by DNA and RNA extraction, the remaining sample, if available, was used to establish 3D tumor-spheres (Fig. 2). Briefly, cells derived from enrichment of BM-infiltrating NB cells, were seeded in 48- and 24-well plates, at a concentration ranging between 5 × 10^5^ and 1 × 10^6^ cells/mL, in tumor-sphere medium, TSM: DMEM-F12/Neurobasal (2:1) medium (Life Technologies), supplemented with 1 × B27 supplement (Life Technologies), 20 ng/ml human recombinant EGF (Life Technologies), 20 ng/ml human recombinant FGF2 (Life Technologies), 50 IU/mL penicillin G (Euroclone), 50 µg/mL streptomycin sulphate (Euroclone), and 2 mM L-glutamine (Euroclone). Under these culture conditions, primary NB cells grew as tumor-spheres, which were maintained in culture for a period lasting between 2 and 5 weeks. When tumor-spheres reached a huge dimension (higher than 500 μm, in diameter), were disaggregated using Accutase reagent (Euroclone), to avoid them undergoing necrotic death. Then, they were seeded again and/or subjected to immunophenotyping, as already described before. In some cases, tumor-spheres were also frozen to create a repository of patient-derived tumor-spheres (Fig. 2). Tumor-spheres were also obtained from tumor cells derived from generations of established PDX and CPDX. In this case, tumor fragments were firstly mechanically reduced to single cell suspensions by the use of BD Medimachine System, as described in the “Flow Cytometry” paragraph. Then cells were cultured, as already mentioned above. Samples grown as tumor-spheres were also frozen by the use of a freezing solution composed of 40% FBS, 50% TSM and 10% DMSO.

### DNA and RNA extraction

Before DNA and RNA extraction, samples were stored as follow: (i) tumor tissue samples were stored at − 20 °C in RNAlater stabilization solution (Qiagen), for both DNA and RNA extraction; (ii) cells, deriving from BM enrichment, tumor-spheres and peripheral blood of the respective patients, were washed in PBS and then, the dry pellet was stored at − 20 °C for DNA extraction; iii) cells deriving from BM enrichment were stored at − 20 °C in RNAlater stabilization solution for RNA extraction.

DNA was extracted from: (I) patient-derived tumor tissue; (II) PDX- and CPDX-derived tumor tissue; (III) single cell pellet of BM-derived NB cells (BM derived from either iliac crests or tumor bone lesions); (IV) patient-derived tumor-spheres; (V) peripheral blood.

Samples were differently processed based on their origin.

For total DNA extraction from tissues and cells, the QIAamp DNA mini or micro kit (QIAgen) was used, respectively, according to the manufacturer’s instruction. Briefly, tumor tissues were first mechanically homogenized using the TissueLyser system (QIAgen) before the addiction of the kit lysis buffers, while cell pellet was directly lysed. Lysates underwent a proteinase K digestion and a RNase A treatment for removal of contaminating RNA. Then, the obtained solution was loaded onto the kit columns and, after washing, the purified DNA was eluted with nuclease-free water. The DNA concentration was quantified through fluorometric assay using Qubit platform (Thermo Fisher Scientific). DNA integrity was preliminary assessed by gel electrophoresis (0.8% agarose). Samples were stored at − 20 °C until use.

RNA was extracted from: (I) patient-derived tumor tissue; (II) PDX- and CPDX-derived tumor tissue; (III) single cell pellet of BM-derived NB cells.

For RNA extraction from tissues and cells, the miRNeasy mini or micro kit (QIAgen) was used, respectively. The kit is designed to extract total RNA combining phenol/guanidine-based lysis and silica membrane–based purification.

RNAlater was removed from tissues and cells and QIAzol lysis reagent (QIAgen) was added to the samples. Tumor samples were mechanically homogenized using the TissueLyser (QIAgen). Then, chloroform was added to the homogenate and after the separation of the phases by centrifugation; the upper aqueous phase containing the nucleic acids was loaded onto miRNeasy mini or micro kit columns and processed following the manufacturer’s instruction. An on-column DNase I treatment was included for the removal of contaminating genomic DNA. Purified RNA was eluted in nuclease-free water and RNA concentration was quantified through absorbance at 260 nm by using Nanodrop instrument. Samples were stored at -80° C until use.

### Whole exome sequencing

#### DNA quantification and library preparation for sequencing

A total of 1.0 µg of DNA per sample was used as input material for library preparation. Genomic DNA was sonicated to a size of 350 bp, and then fragments were end-polished, A-tailed, and ligated with the full-length adapter for Illumina sequencing with further PCR amplification. At last, PCR products were purified (AMPure XP system) and libraries were analyzed for size distribution using the DNA Nano 6000 Assay Kit of Agilent Bioanalyzer 2100 system (Agilent Technologies, CA, USA) and quantified using real-time PCR. The Agilent SureSelect Human All Exome V6 was used to capture exome regions. A total of five samples, with low tumor cellularity (< 60%), were sequenced using a panel of 484 cancer-related genes (CGP; Cancer Gene Panel) generated by xGen™ Custom Hybridization Capture Panels (Integrated DNA Technologies, CA, USA). The DNA sequencing produced paired-end reads of 150 bp.

#### Alignment and variant calling

Mapping BAM files were obtained with BWA and SAMTools [20, 21] by aligning the sequencing reads versus the GRCh37/hg19 reference genome assembly. Somatic Single Nucleotide variants (SNVs) and small insertions and deletions (INDELs) were detected with GATK MuTect2 [22]. Germline SNVs and INDELs were detected with GATK HaplotypeCaller. The FREEC and AnnotSV programs were used for the identification and functional annotation of the CNVs. The functional annotation of somatic and germline variants was performed with ANNOVAR [23]. An additional variant calling was performed by adding the WES data from the 3D samples.

#### Quality control of the sequencing

A total of 50 samples were sequenced: 18 tumor samples, one independent consecutive relapsed tumor (Pz#2bis) from Pz#2 and 17 matched peripheral blood samples (for Pz#1 the germline DNA was not available), 10 PDX and CDX samples, and 4 tumor-sphere 3D models (Table 1). Overall, we collected high quality sequencing data with, on average, the 51.63 millions of reads (75.15 and 48.69 million for CGP and WES, respectively) (Additional file 1: Figure S1A). The percentage of bases with quality scores above 30 (Q30) was 93.70% (93.09% and 93.78% for CGP and WES, respectively) (Additional file 1: Figure S1B). After the alignment, the mapping rate was of 99.86% (99.68% and 99.88% for CGP and WES, respectively) (Additional file 1: Figure S1C). The duplicate rate was of 22.03% (29.24% and 21.13% for CGP and WES, respectively) (Additional file 1: Figure S1D). We obtained an average sequencing depth of 290x: 129 × for normal controls and 387 × for tumors (1611 × and 183 × for CGP and WES, respectively) (Additional file 1: Figure S1E). Overall, the 99.59% of the target region was covered (99.90% and 99.55% for CGP and WES, respectively) (Additional file 1: Figure S1F). Furthermore, the fraction of the target covered with at least 10 and 50 reads, was of 98.10% (99.64% and 97.91% for CGP and WES, respectively), and of 84.13% (99.20% and 82.25% for CGP and WES, respectively) (Additional file 1: Figure S1G, H). These results allowed us to reliably detect both germline and somatic variants.

#### Variant filtration

From raw calls, we first discarded SNVs and INDELs that did not pass MuTect2 or HaplotypeCaller quality filters [24]. To remove possible false positives, we eliminated variants falling in genomic duplicated regions. We filtered out common polymorphisms (minor allele frequency > 1%) by using allele frequencies of non-Finnish European populations in 1000 Genomes Project, ExAC and gnomAD databases [25, 26] and removed off-target mutations. The set of exonic variants was then filtered to remove synonymous SNVs. Since germline DNA was not available for the Pz#1 sample, we filtered out the variants with the population maximum allele frequency greater than 0.001 and not included in the COSMIC database.

**Germline variants** were restricted to a list of cancer predisposition genes published in [11]. Moreover, we further analyzed variants annotated as “Pathogenic” or “Likely pathogenic” in ClinVar or InterVar (American College of Medical Genetics) databases. Germline variants were excluded if annotated in ClinVar with “conflicting interpretations”.

**Somatic variants** were excluded if supported by less than 8 reads and with a variant allele frequency below the 5%. Finally, somatic variants were prioritized if they had the pathogenic CADD score (vl.6) [27] greater than 20 or CancerVar [28] greater than 0.80.

Regarding the CNVs selection, biallelic deletion (CN = 0) and focal amplifications (CN ≥ 10) were considered for tumor suppressor genes and for oncogenes, respectively. We also excluded the CNVs categorized as benign according to ACMG classification [29] and those that did not pass the quality check performed by visual inspection of the Integrative Genome View (IGV) file.

#### Cancer gene panel Tumor Mutational Burden (TMB) calculation

TMB was calculated from the count of missense variants detected from the panel with a variant allele frequency (VAF) of 5% or higher. The count was divided by the length of targeted (non-overlapping) regions (~ 3.04 Mb; 3,040,053 bp) to give a value in mutations per Mb. TMB values of ≥ 5 were considered actionable based on trial eligibility (NCT02992964).

#### Exome TMB calculation

TMB was calculated from the count of missense variants detected in the exome with a VAF of 5% or higher. This count was divided by the length of targeted (non-overlapping) regions (~ 38.29 Mb; 38,289,292 bp).

#### Microsatellite instability (MSI)/mismatch repair (MMR) calculation

MSI status was computed as previously published [30]. MSI score of 10 or higher was defined as MSI high and potentially targetable with immune checkpoint inhibitors.

#### Somatic variants in PDX and CPDX generations and in 3D samples

Five samples with the corresponding tumors grown up to the second (P2) generation were collected; for four of these samples, 3D models grown from tumor cells derived from the PDX or CPDX P2 generation were also obtained. These samples underwent WES and somatic variants were called and filtered as above described. To evaluate the grade of genomic similarity among original patient-derived tumors and their respective derived tumor models (PDX/CPDX generations and 3D cells), Pearson correlation, Clustering analysis (Euclidean distance and Ward’s method) and Principal Component Analysis (PCA) were performed, using the variant position and the allele frequencies of all mutations detected by WES. Then, the sets of filtered variants (minor allele frequency > 1% and missense changes) were used to get counts of conserved variants across PDX/CPDX generations and 3D models.

### RNA sequencing

#### Library preparation

RNA-Seq analysis was performed on 15 tumor samples: 9 NB relapsed tumors, 1 CPDX (from Pz#C) and 3 PDX (from Pz#3, Pz#4 and Pz #8) samples at first generation (P1) and 2 PDX (from Pz#3 and Pz#4) samples at second generation (P2) (Table 1).

Libraries were prepared using the Illumina QuantSeq 3’mRNA-Seq FWD Library Prep Kit following the manufacturer’s instructions. RNA integrity and sample concentration were then checked with an Agilent 2100 Bioanalyzer system (RIN > 10, 100 ng/ul on average). Libraries were pooled, and sequencing runs were performed in single-end mode using the Illumina NovaSeq6000 platform generating 15 million reads per sample on average.

#### Quality control, alignment and post-processing

Initial RNAseq quality control checks included evaluating the sequence quality scores, GC content, sequence read length and adapter contamination using FastQC (v 0.11.5). Universal Illumina adapter sequences (AGATCGGAAGAG) were removed with Cutadapt (v3.4), and read trimming was performed with Trimmomatic (v 0.39) with parameters HEADCROP = 10, MINLEN = 50, AVGQUAL = 30. Reads were then aligned to the human genome assembly (build hg38) using STAR (v. 2.7.8a), two-pass method and quantMode parameters set to TranscriptomeSAM for alignments translated into transcript coordinates. Post-alignment QC required at least 70% of reads to be uniquely aligned, assessed using STAR alignment statistics (100% of samples passed). Post-processing included sorting and filtering per alignment quality (MAPQ > 30) with Samtools (v.1.7).

#### Data analysis

For each tumor, from 10 to 20 million sequencing reads (except for one sample with more than 30 million reads) and an average alignment rate of 80% (Additional file 1: Figure S2A, B) were obtained. Feature counting was performed with the R package FeatureCount [31] and genes with less than 20 reads detected in all the samples were filtered out. Raw read counts were normalized with the CPM method using the R package EdgeR [32]. The grade of genomic similarity among samples were evaluated by Spearman correlation, Clustering analysis (Euclidean distance and Ward’s method) and PCA. We used the R package GeneOverlap to study if the overexpressed genes were maintained across the different tumor generations. To identify over-expressed genes in each tumor sample, we computed for each sample the Z-score of the log2 CPM values and retained the genes with Z-score equal or greater than 3. Fusion genes were detected using STAR-fusion (v1.11.0) with default parameters https://github.com/STAR-Fusion/STAR-Fusion/wiki.

#### Selection of potentially therapeutically targetable somatic alterations

Each genetic alterations were manually curated with the support of diverse public databases: Clinical Interpretation of Variants in Cancer (CIViC) (https://civicdb.org/welcome) and Oncology Knowledge Base (OncoKB) (https://www.oncokb.org/) that contain biological and clinical information of genomic alterations, and Drug Gene Interaction Database (DGIdb) (https://www.dgidb.org/) and Therapeutic Target Database (TTD) (https://db.idrblab.net/ttd/) that collect information on drugs, target genes and clinical trials.

The selection of potentially actionable genetic alteration related to CNVs was based only on targets with approved agents or in clinical trials (Additional file 2: Table S1).

The actionable genetic alterations were categorized according to two grades of priority as follows:

“Very high priority” - molecular alterations that are validated to be directly targetable by an already approved drug or an investigational agent in any phase of clinical development;

“High priority”- novel molecular alterations that are predicted to be pathogenic in therapeutically actionable genes or pathways targetable by already approved drugs or an investigational agent in any phase of clinical development.

Molecular alterations were excluded if the action mechanism (inhibitor or antagonist) of the drug did not correspond with the action mechanism of mutations (loss-of-function and gain-of-function) in relation to the gene function (tumor suppressor and oncogene).

#### Therapeutic recommendations of Molecular Tumor Board (MTB)

The criteria to suggest the targeted treatment were based on the ESMO scale for Clinical Actionability of molecular Targets (ESCAT) [33], adapting the evidence scale to the pediatric population. The MTB focused the discussion by considering clinical evidences, ongoing clinical trials and previous treatments received by the patient, and by defining the somatic mutation(s) as targetable by an approved or investigational agent, either directly or indirectly in the affected pathway.

## Results

### NB patients evidenced one or more potentially actionable genetic alterations

Among the 17 samples with available germline sequence results (Additional file 2: Table S2), two samples (11.7%) exhibited germline pathogenic variants in known cancer predisposition genes. The identified variants were located in *MITF* (p.E318K) and *TSHR* (p.P162A), and detailed descriptions can be found in Additional file 2: Table S3. All reported pathogenic variants were heterozygous, and no somatic events were detected in the corresponding tumors.

**Fig. 3:**
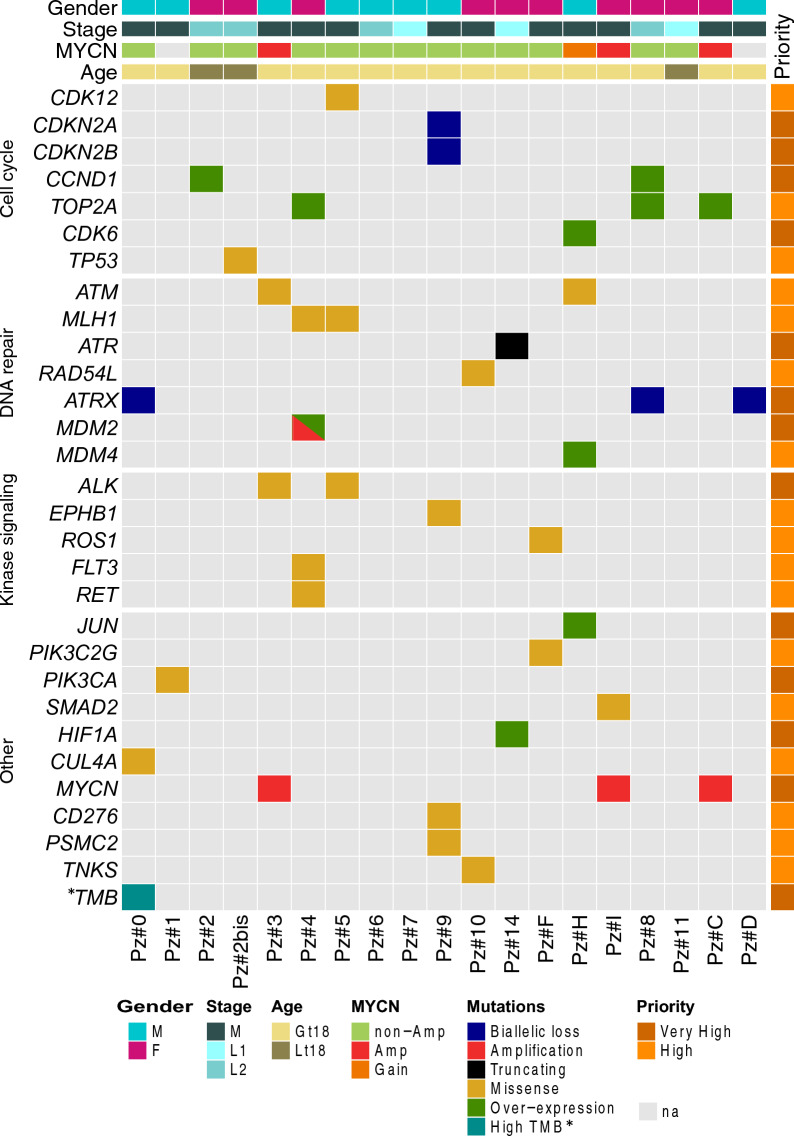
Summary view of the potential therapeutically targetable somatic alterations. The data matrix shows the targetable pathogenic somatic variants detected in relapsed tumors (Pz). Mutated genes are reported by row, and samples are reported by column. On the top of the matrix, we reported the patient characteristics (gender, disease stage, MYCN amplification status and the age at diagnosis; *Gt18* age at diagnosis ≥ 18mo, *Lt18* age at diagnosis < 18mo). The matrix is split by rows based on the involved pathways (source: MyCancerGenome). The annotation track on the right reports the level of priority of the variant based on the pathogenicity and actionability (see Methods). TMB*: Tumor Mutational Burden. High TMB was considered if the patient had more than five variants per megabase of sequencing target regions

After the filtering of somatic SNV/INDELs and CNVs, (see Methods), we retained a total of 540 SNV/INDELs (Additional file 2: Table S4) and 58 CNVs (Additional file 2: Table S5) classified as pathogenic that were then considered for further evaluation to establish their potential clinical actionability according to the criteria listed in the Methods. In total, 16 (84%) of 19 processed NBs from 18 patients (for the patient Pz#2, two independent consecutive relapsed tumors were analyzed) had at least one potentially therapeutically targetable somatic alteration from comprehensive sequencing including DNAseq and RNAseq data (Fig. 3 and Additional file 2: Table S6). These 40 events included SNVs/INDELs (n = 21, 53%), CNVs (n = 8, 20%), overexpressed genes (n = 10, 25%), and hypermutation (n = 1, 2%). No findings resulted from the analysis of MSI status and gene fusions. The most frequently affected among druggable pathways included DNA damage/repair (31%), cell cycle control (28%) and receptor tyrosine kinase/growth factor signaling (19%). We found 19 targetable alterations categorized as “very high priority” in 13 out of 19 tumors (68%): 8 CNVs (42%), 6 gene expression outliers (32%), 4 SNVs (21%) and 1 hypermutation (5%). Clinically actionable alterations known to be recurrent in NB were found: *MYCN* amplification (n = 3, 16%) [2], *CDK6* or *CCND1* over-expression (n = 3, 16%) [34–36], *ATRX* loss (n = 3, 16%) [37], *ALK* point mutations (n = 2, 11%) [5] and *MDM2* amplification (n = 1, 5%) [38]. One sample carried the H1047R hotspot mutation in the *PIK3CA* gene reported to be an actionable target in diverse tumors [39]. In another tumor, we observed the over-expression of *HIF-1A* gene that has been reported in many cancers and for which diverse agents are currently being developed or are under investigation in clinical studies [40].

### Therapeutic recommendations

A genetic counseling was suggested by the MTB to two patients carrying cancer predisposing germline variants. Treatment recommendations were instead given for 10 patients, and 4 of them (Pz#0, Pz#2, Pz#9, Pz#10) received the target drug as single agents or associated with chemotherapy for 4 different mutations (CUL4A, TP53, PMSC2 and TNKS, respectively). The patients with *CUL4A* and *PSMC2* genes mutation have been already reported [41]. The patient with TP53 mutation, an infant L2 at diagnosis, was enrolled in PREME after the fourth relapse. According to the genomic profile results and to previous treatments received, MTB proposed a combination treatment with Irinotecan-Bortezomib. After 2 cycles, patient rapidly progressed again (abdominal progression with effusion), which led to the suspended therapy to start palliative oral chemotherapy. The patient with TNKS mutation received therapy with the TNKS inhibitor, Stenoparib-2 × 121, as compassionate use. However, after 2 weeks of treatment, the patient experienced a skin toxicity of grade 3 with extensive maculo-papular rash associated to photosensitivity. The patient showed also sign of PD (periorbital ptosis edema and left eyelid ecchymosis), which led to the suspended Stenoparib-2 × 121 therapy to start standard chemotherapy. The other 6 patients with potentially therapeutically targetable somatic alterations, and recommended for treatment by the MTB, did not receive the suggested treatment either because in complete remission following second line therapy, or because in progressive disease or already dead before treatments. The other 6 patients with potentially therapeutically targetable somatic alterations were not discussed by the MTB either because the genomic results were obtained after patients death, or because in effective ongoing therapy.

### The genetic, histological, immunophenotypic and transcriptomic profiles of PDX and CPDX models were highly conserved compared to the original tumors

Four out of 10 tumor samples implanted into mice (40%) (Table 1), (deriving from Pz#3, #4, #5 and #8) grew subcutaneously, becoming the first generation (P1) of PDX (PDX#3P1, #4P1, #5P1 and #8P1, respectively). Tumor growth lasted from 3 to 8 months after implantation, confirming the long latency of tumor formation [42]. The only inoculated tumor sample derived from BM-infiltrating NB cells (Pz#C), led to the growth of a tumor 2.5 months after cells injection, becoming the first generation of CPDX (CPDX#C-P1). To date, the tumor take and growth seem to be independent from tumor stage and *MYCN* status (Table 1). PDX and CPDX models were then expanded to the second generation (P2). The histological and the cytometric (when the biological materials were available) immunophenotyping evaluations of the P1 and P2 tumor generations confirmed the maintenance of almost all the biological features of the original patient-derived tumors (Table 1).

**Fig. 4:**
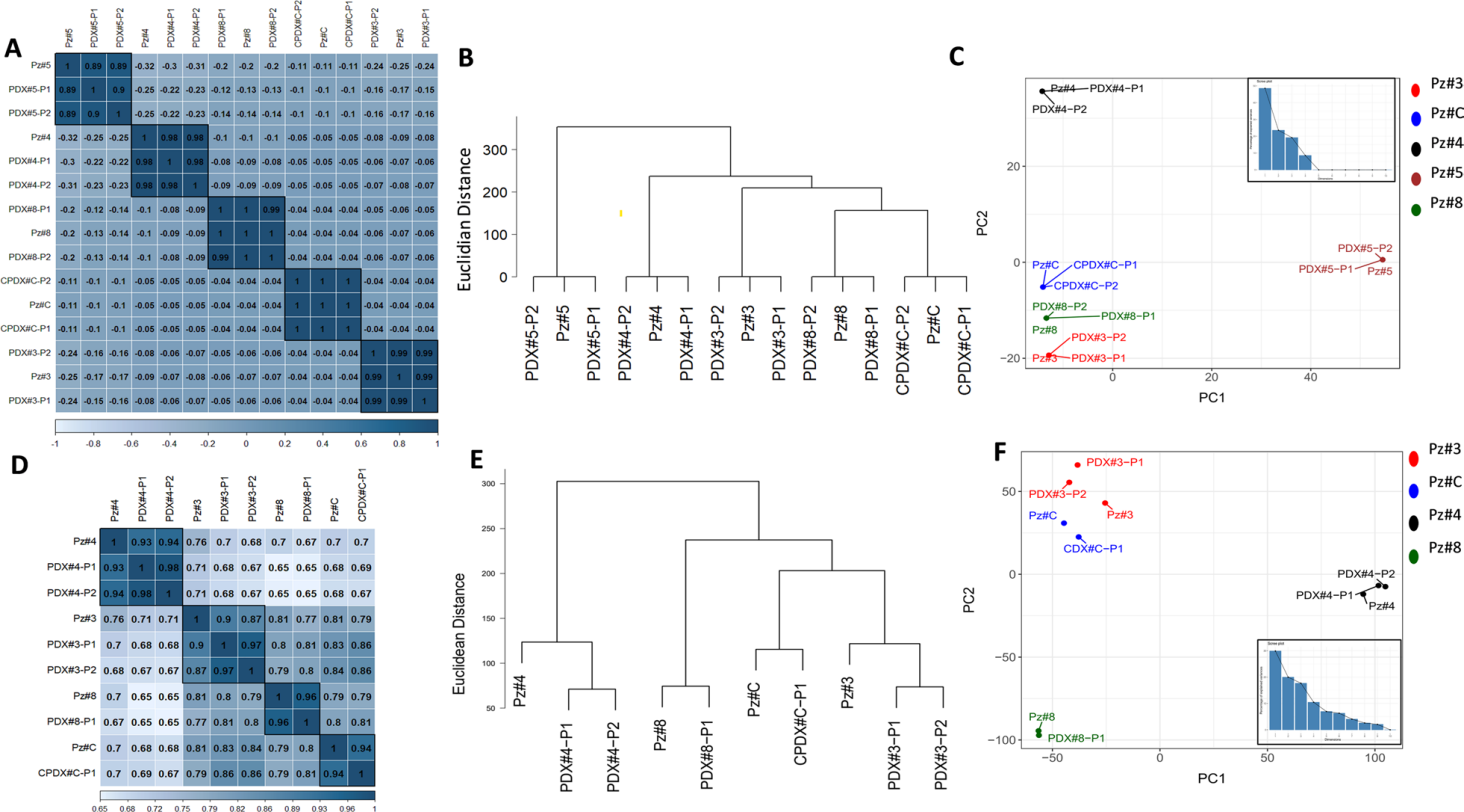
Comparison of genetic variations and transcriptomic profiles among patient’s tumors and PDX or CPDX generations. For each analysis, tumors at first (P1) and second (P2) generations cluster together with the respective original patient tumors (Pz#). **A**–**D** Clustered heatmap of the correlation coefficients for SNVs and gene expression levels, respectively. **B**–**E** Hierarchical clustering using Euclidian distance and Ward’s method for SNVs and gene expression levels, respectively. **C**–**F** Principal Component Analysis and Scree Plot (bottom right) for SNVs and gene expression levels, respectively

Exome sequencing data were applied to assess the genomic similarity among relapsed patient tumors and their corresponding PDX or CPDX samples up to second generations (n = 15 samples, Table 1). Pairwise Pearson correlation, clustering and PCA analyses using all the detected SNVs (without filtering) were performed. Overall, the results of our comparative analyses showed a high grade of genetic similarity among the originating tumors and their corresponding P1 and P2 tumor counterparts developed in mice (Fig. 4A–C).

The VAF median increased at P1 and P2 generations (P < 1 × 10^–15^ Mann–Whitney test) when compared to the matched original tumors (Additional file 1: Figure S3). The difference of VAF median between P1 and P2 generations was instead less marked but remained statistically significant (P < 1 × 10^–15^ Mann–Whitney test) (Additional file 1: Figure S3). In a subsequent analysis, we focused on the selected rare and missense somatic variants to count the number of variants that were conserved among murine xenograft generations. We observed that at least more than 77% of somatic variants were conserved at first generation and at least more than 84% at second one compared to the original patient tumors for all samples except for Pz#4 (Additional file 2: Table S7).

**Fig. 5:**
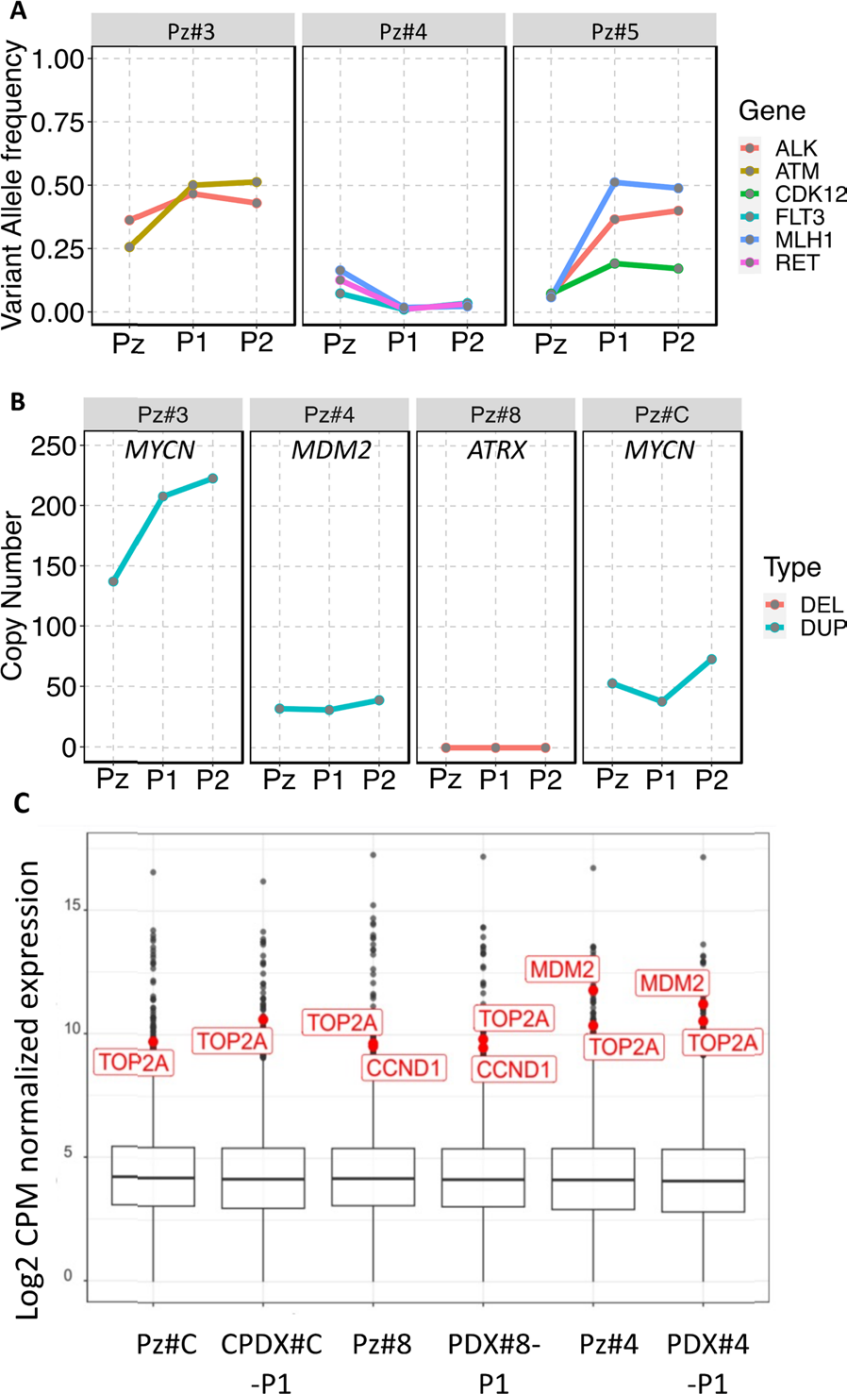
Tracking of potential therapeutically targetable somatic alterations among PDX or CPDX generations. **A** The figure shows the allele frequency of actionable pathogenic somatic variants detected in patient’s tumors and tracked in murine xenografts generations. **B** The figure shows the number of copies of actionable pathogenic somatic copy number variants detected in patient’s tumors and tracked in murine xenografts generations. *Pz* patient’s tumor, *Pl *first PDX generation, *P2* second PDX generation, *DEL* Deletion; *DUP* Duplication, **C** Box plots showing the expression of gene levels of patient’s tumors and murine xenografts generations. The targetable gene showing an abnormal over-expression is reported in the red box

Finally, we wanted to assess if the potentially therapeutically targetable genetic alterations found in relapsed tumors from Pz#3, Pz#4 and Pz#5 (Fig. 3 and Additional file 2: Table S6) were conserved in the P1 and P2 generations. All SNVs were conserved in the PDX/CPDX models (Fig. 5A). Particularly, a VAF increase of actionable SNVs in Pz#3 and Pz#5 moving from patient’s tumors to P1 generation was observed. Instead, from P1 to P2 generations, the VAF was almost stabilized (Fig. 5A). In contrast, the VAF of actionable SNVs in Pz#4 tended to decrease in the subsequent two PDX generations (Fig. 5A). All the actionable CNVs observed in tumors were conserved in the first and second generation of PDX/CPDX models with an increase of copy numbers (Fig. 5B**)**.

We also verified whether the overall transcriptomic profile was maintained across the different generations of PDX and CPDX models. For this purpose, we computed the pairwise Spearman correlation coefficients of the sample’s gene expression. Overall, we found that the expression profile of each original patient tumor was highly correlated with its xenograft generations (Fig. 4D). The P1 and P2 generations of each tumor were more similar among themselves than the original derived tumor (Pearson correlation in the range 0.97–1), suggesting cell population selection in the homing of the tumor explants in the mice. Similar results were obtained using two orthogonal approaches, the hierarchical clustering approach based on the Euclidian distance and the PCA (Fig. 4E–F). We also confirmed the identification of all gene expression outliers in murine xenograft generations when available (Fig. 5C).

Altogether, the obtained results suggest that genomic features of tumors are conserved in the P1 and P2 generations of xenograft tumors. Furthermore, once the maintenance of the histopathologic and immunophenotypic characteristics of the original patient tumor was demonstrated in the murine generations (Fig. 2), a repository of PDX and CPDX was created for subsequent therapeutic applications.

### The genetic and immunophenotypic profiles of patient-derived 3D/tumor-spheres were highly conserved compared to the original tumors and to the PDX—CPDX models

3D models were developed using PDX#3, PDX#4, PDX#5 and PDX#8 samples obtained at P2 generation (here named P2-3D). For the Pz#C, we were able to generate 3D sphere models from the original patient BM-infiltrating NB cells and from the P1 and P2 generation of PDX tumors (CPDX#C-P1 and CPDX#C-P2). All the samples utilized grew in the 3D conditions (Additional file 1: Figure S4). Tumor-spheres derived from PDX/CPDX were also cultured after thawing. They were still viable, as assessed by trypan blue exclusion dye, and when cultured in tumor-sphere medium, were able to grow again as tumor-spheres (data not shown). Tumor-spheres derived from the different samples, had similar morphological characteristics, in term of shape, all of them creating sphere-like structures (round-to-oval, Additional file 1: Figure S4). Nevertheless, they differed from each other for their compactness. Indeed, by comparing the 3D structures, it seems evident that some were organized in a compact tumor-spheroids (Additional file 1: Figure S4A, PDX#3, PDX#4 and CPDX#C), while others appear as tight aggregates (Additional file 1: Figure S4B, PDX#5, PDX#8), suggesting a different adhesion molecules expression profile [43, 44]. Moreover, with the intention of reducing the number of animals potentially enrolled in future preclinical studies (in accordance to the 3R rules), and to consequently use alternative preclinical models, as a proof of concept, 3D deriving from cells of the different generation of CPDX#C were developed in the same medium and compared. As illustrated in Additional file 1: Figure S4C, the tumor-spheres derived from Pz#C and from the subsequent CPDX#C-P1 and P2 generations, had comparable shape and compactness.

From an immunophenotyping point of view, based on the analyses performed on the samples characterized when available, it was apparent that all the classic NB markers (CD56, GD2, B7-H3) and the recently discovered cell surface receptor nucleolin (NCL) [45], are maintained along the different PDX generations and the PDX-derived 3D spheroids. We then conducted an exome sequencing of 3D models obtained from P2 PDX generation with the purpose of assessing the grade of genetic similarity with their matched patient tumors, and subsequent murine xenograft P1 and P2 from which they were generated. Using the somatic SNVs of each sample (n = 16, Table 1), we performed analyses of pairwise Pearson correlation, clustering and PCA to assess their level of genetic similarity. (Additional file 1: Figure S5A–C). Overall, a high grade of similarity between the P2 sample and its 3D counterpart was observed, as evidenced by the results from Pearson correlation analysis (Additional file 1: Figure S5A). Of note, we also observed a high genetic similarity among the P2-3D models and their matched patient tumors and P1 generation. We observed a significant decrease (P < 1 × 10^–15^; Mann–Whitney test) of allele frequencies for variants detected at P2-3D when compared to P2 (Additional file 1: Figure S5D). We then focused on the rare and missense somatic variants to count the number of variants that were conserved among tumor generations and the 3D generated from P2 murine tumor xenografts. We observed (Additional file 2: Table S7) that at least more than 52% and 46% of somatic variants, respectively, were conserved at P2-3D compared to P2 and P1 tumors, respectively, except for Pz#4. Finally, the presence of somatic potentially actionable variants found in relapsed tumors from Pz#3 and Pz#4 and in the corresponding P2-3D samples was also assessed. A decrease of VAF for 4 out of 5 variants moving from P2 to their 3D models was seen (Additional file 1: Figure S6A). The therapeutically actionable CNVs were also conserved in 3D models (Additional file 1: Figure S6B).

The results obtained in 3D models recapitulate the genomic and histologic features of the tumors of origin, suggesting that they can represent a valid alternative to PDX animal models for understanding cancer biology preclinical studies.

## Discussion

In this manuscript, we present the findings of the Italian personalized medicine program, PREME, designed specifically for patients affected by neuroblastoma (NB). Through this program, we identify NB patient samples that carry targetable genomic alterations, enabling the establishment of Patient Derived Xenografts (PDX), Cell PDX (CPDX) and 3D repositories. These resources prove to be valuable assets for translational research, offering promising avenues for advancing our understanding and treatment of NB. PREME demonstrates that comprehensive molecular analysis of samples from NB patients is feasible on a national scale and that the generated preclinical models (PDX and 3D tumor-spheres, derived from both NB tumors and bone marrow-infiltrating NB cells), recapitulate histological and genomic features of the originating tumors.

Considering the possible clinical impact of detected variants, 16 out of 19 processed tumors had 1 or more alteration with potential therapeutic implications. Thirteen (68%) of these samples were validated to be directly targetable by an already approved drug or an investigational agent. Our data are in line with recent results of other precision medicine programs for pediatric cancers, considering that our sample cohort includes 95% (18 out of 19) of relapsed tumors, reported to have a higher number of targetable alterations respect to the primary ones [44]. Indeed, INFORM [16], iTHER [14], Zero Childhood Cancer Program[13], MAPPYACT [12], GAIN/iCat2 [15] and Villani et al. [17] have reported therapeutically targetable mutations in 85.9%, 81.9%, 71.4%, 69%, 65% and 54% of analyzed patients, respectively. However, it should be considered that there is still a discrepancy in the rate of actionable mutations among the precision medicine programs, probably because a definition and prioritization of actionable events have not been standardized and harmonized in pediatric oncology, as compared to adult oncology. By exome sequencing and FISH approaches, we detected the most known NB recurrent alterations: the amplification of *MYCN* (n = 3.16%) [2], *ATRX* loss (n = 3.16%) [37], *ALK* point mutations (n = 2.11%) [5] and *MDM2* amplification (n = 1.5%) [38]. These data demonstrate the reliability of our sequencing and bioinformatic methods. Interestingly, in this work the most frequent type of alterations observed and categorized as “very high priority” are represented by CNVs (42%), confirming that NB is mainly characterized by pathogenic and clinically relevant chromosomal aberrations. Indeed, the observed loss of *ATRX* can be therapeutically exploited by a combination of olaparib and irinotecan in NB [46], while *CDKN2A* and *CDKN2B* biallelic loss and *MDM2* amplification, found in two distinct tumors, are potentially targetable by different small molecule inhibitors that are currently being tested in clinical trials [47, 48]. Furthermore, we also observed a high rate of gene expression outliers, shown to be effective therapeutic targets in adult [49] and pediatric cancers [50]. Notably, we observed the over-expression of *TOP2A*, a potential chemotherapeutic target [51], *CCND1*, a potential target for CDK4/6 inhibitors with ongoing clinical studies in NB patients (NCT05429502, NCT01747876) and *MDM2*, which has attracted attention with the development of numerous small molecule inhibitors [52]. Over-expression of these genes was also confirmed in murine xenograft generations. On the contrary, no gene fusion was found, suggesting a paucity of this type of molecular alterations in NB.

Numerous patients exhibited multiple potential actionable oncogenic events across various cancer hallmarks, highlighting the significant complexity of cancer that current clinical trials have not fully addressed yet. This observation underscores the need for further refinement and adaptation of clinical trial designs to effectively target and treat the intricate landscape of cancer-associated mutations and pathways. The findings emphasize the importance of advancing personalized medicine approaches to better tailor treatments to individual patients' specific genomic profiles and overcome the challenges posed by cancer heterogeneity.

It is known that higher TMB results in more neo-antigens expression, thus increasing the chances for T-cell recognition. Indeed, TMB is clinically associated with more favorable outcomes after treatment with immune checkpoint inhibitors [52]. Our analysis of TMB showed that NB, as most of the childhood malignancies [53], present a low rate of somatic point mutations; indeed, only one patient showed a high TMB. This result indicates that, in NB, TMB can be considered as a rare biomarker to predict immunotherapy response. The same observation can be extended to the MSI results, being MSI another indicator of sensitivity to immunotherapy [54]. Indeed, no tumors showed a significant increase of the number of microsatellites.

In PREME, the rate of pathogenic germline variants in cancer predisposition genes was 12%, in accordance with the results obtained in our very recent exome sequencing study including many other NB patients [11]. We here found a mutation in *MITF* gene, which is recurrent in familial and sporadic melanoma [55] and, more interestingly, has been previously reported in three additional NB patients [56, 57]. These results further support the relevance of performing germline sequencing for NB patients at diagnosis to address a genetic counseling.

To note, eighty-four % of the evaluated NBs had at least one potential therapeutically targetable somatic alteration. Treatment recommendations were given by the MTB for 10 patients, and 4 of them received the target drug as single agents for 4 different mutations, or associated with chemotherapy. The discrepancy between the number of actionable targets identified and the number of patients treated accordingly might be explained by the definition of “actionable”, which does not take into account the availability of drug or trial for pediatric patients. Moreover, some patients did not receive the matched treatment because of a declining clinical outcome or because already enrolled in effective treatments.

Xenografts obtained by the direct implantation of either tumor fragments or cell suspensions derived from patient’s tumors into immunodeficient or humanized mice [58] have emerged as an important tool for translational research. In case of metastatic and/or relapsed NB, the main limitation faced to set up PDX, is represented by the difficulty to access enough fresh tissue. In such a case, the possibility to isolate BM-infiltrating NB cells from either iliac crests or tumor bone lesions might represent an interesting option for the establishment of CPDX. As previously suggested by Tucker E.R. and colleagues [42], a CPDX was here developed, for the first time to our acknowledgement, from BM-infiltrating NB cells, and subsequent CPDX generations histologically and genomically characterized. Furthermore, the enrichment of BM-infiltrating NB cells to rule out as much CD45^pos^ cells contaminating the tumor samples as possible, could potentially lead to an increased rate of “PDX” tumor growth. The PDX and CPDX models here generated, maintained cellular and histological structure of the original tumors, also including critical stromal elements. Moreover, molecular analysis of tumors from PDX or CPDX up to the second generation revealed a strong preservation of somatic mutations and gene expression profiles of the corresponding patient tumors. Importantly, the candidate targetable gene expression outliers, SNVs and CNVs, found in patient tumors, were conserved in the first and second generation of xenograft models. Interestingly, by comparison to patient tumors, consistent with a previous study [59], we found that VAFs in PDX/CPDX were overall higher, which reflects high tumor purity.

Patient-derived tumor-spheres also represent an excellent tool for the high-scale evaluation of drug-efficacy. Furthermore, they can also contribute to the fulfillment of the 3R rules. Since the mutational profiles of tumor-spheres resulted preserved compared to the original tumor, and among successive generations, 3D tumor-spheres derived directly from tumor cells of NB patients, and from tumor cells derived from PDX generations, seem to represent a good alternative to the use of PDX for drug screening [60].

In conclusion, PREME has further demonstrated that comprehensive molecular analysis contributes significantly to the identification of germline and somatic driver variants and to clinical therapeutic recommendations in NBs. Currently, Italian patients with high-risk, relapsed or refractory NB are offered WES and RNAseq in PREME. A pilot study to evaluate the benefit of receiving whole genome sequencing (WGS) rather than WES is ongoing. PREME also demonstrated the feasibility of generating faithful patient-specific preclinical models. NB patient-derived samples xenografted in living zebrafish [61] will be developed and tested in the near future, to reveal PREME-driven precision medicine effects on NB cell dissemination in vivo and to seek for the best pharmacological strategy to be applied in more complex animal models. At present, PDX, CPDX and 3D tumor-spheres from NB tumors were developed and characterized, thus creating, an important resource for the identification of more druggable genetic mutations and discovery of further insights into PDX recapitulation of human tumors.

### Supplementary Information

**Additional file 1: Figure S1.** Descriptive statistics of sequencing and mapping results from 50 samples **A** Box plot reporting the number of sequencing reads used for the alignment. **B** The percentage of bases with quality score above 30 (Q30). **C** The percentage of mapped reads (mapping rate). **D** The percentage of duplicated reads. **E** Box plot reporting the average sequencing depth on target obtained after the alignment and duplicates removal. **F** Box plot reporting the average coverage of target regions. **G** The percentage of target regions covered with at least 10 reads. **H** The percentage of target regions covered with at least 50 reads. The data values in panels **A** and **E** are shown by sample type and by sequencing panel. The data values in panels **B**, **C**, **D**, **F**, **G** and **H** are shown by sequencing panel type. *Pz* relapsed tumor. *P1* first PDX/CPDX generation, *P2* second PDX/CPDX generation. *P2-3D* 3D model derived from the second PDX/CPDX generation. **Figure S2.** Read counts and alignment statistics of RNAseq analysis. The figure summarizes the read counts and alignment statistics of each sample. **A** For each sample, we obtained from 10 to 20 million sequencing reads on average except for the sample Pz#C with more than 30 million reads. **B** On average, 80% of total reads were uniquely mapped to the genome, and 16% were mapped to multiple loci. **Figure S3.** Comparison of variant allele frequency among patient’s tumors and PDX/CPDX generations. Box plot showing the distribution of the variant allele frequency. *Pz* patient’s tumor, *Pl* first PDX/CPDX generation, *P2* second PDX/CPDX generation. The values of each patient are represented as dots on each boxplot, and they are distinguishable based on the color code provided on the right side of the figure. Each color corresponds to a specific group or category of patients, allowing for easy identification and comparison of data across different groups. **Figure S4.** 3D models. The figure shows representative pictures of 3D/patient-derived tumor-spheres, developed by using either tumor cells derived from first (P1) and second (P2) generation of PDX and CPDX or tumor cells directly derived from patient (Pz#C). **A** Compact tumor-spheroids derived from PDX#3, PDX#4 and CPDX#C all at P2 generation. **B** 3D structures forming tight aggregates derived from PDX#5 and PDX#8 at P2 generation. C Tumor-spheres of patient C and subsequent generations P1 and P2. Bar: 100 µm. **Figure S5.** Comparison of genomic variations between patient’s tumors, PDX/CPDX generations and 3D models. The figure shows the results of analysis based on the variant allele frequencies of all the detected variants in the group of four tumors having PDX/CPDX samples up to second generation and 3D model production. *Pz* patient’s tumors. *P1* first PDX/CPDX generation. *P2* second PDX/CPDX generation, *P2-3D*, 3D model grown from the second murine xenograft generation. **A** Clustered heatmap of the correlation coefficients. **B** Hierarchical clustering using Euclidian distance and Ward’s method. **C** Principal Component Analysis and Scree Plot (bottom right). **D** Box plot showing the distribution of the variant allele frequency. The values of each patient are represented as dots on each boxplot, and they are distinguishable based on the color code provided on the right side of the figure. Each color corresponds to a specific group or category of patients, allowing for easy identification and comparison of data across different groups. **Figure S6.** Tracking of potential therapeutically targetable somatic alterations in PDX or CPDX generations and 3D models. **A** The figure shows the allele frequency of targetable somatic variants detected in patient’s tumors and tracked in murine xenografts generations and 3D models derived from the second tumor generation. **B** The figure shows the number of copies of potentially targetable somatic copy number variants detected in in patient’s tumors and tracked in murine xenografts generations and 3D models derived from the second tumor generation. *Pz* primary tumor, *P1* first PDX/CPDX generation, *P2* second PDX/CPDX generation.

**Additional file 2: Table S1.** Potentially actionable copy number variants. **Table S2.** Germline pathogenic variants. **Table S3.** Functional roles of genes harboring germline pathogenic variants. **Table S4.** Somatic pathogenic single nucleotide variants and insertions/deletions. **Table S5.** Somatic copy number variants (CN=0 and CN>10). **Table 6.** Potentially therapeutically targetable somatic alterations. **Table S7.** Percentage of conserved somatic variants among patient's tumors and PDX/CPDX and 3D models.

## Data Availability

Still ongoing, no data available yet.

## Abbreviations

NB: Neuroblastoma
PREME: PeRsonalizEd MEdicine program
BM: Bone Marrow
FCM: Flow Cytometry
IHC: Immunohistochemistry
PDX: Patient-Derived Xenografts
CPDX: Cell Patient-Derived Xenograft
NSG: NOD/SCID/IL2Rgamma^null^ (NSG) mice
3D: Three-dimensional models
NGS: Next Generation Sequencing
WES: Whole Exome Sequencing
WGS: Whole Genome Sequencing
CGP: Cancer Gene Panel
FISH: Fluorescence In Situ Hybridization
SNV: Single Nucleotide Variant
INDEL: Small INsertion and DELetion
CNV: Copy Number Variation
TMB: Tumor Mutational Burden
VAF: Variant Allele Frequency
MSI: Microsatellite Instability
MMR: MisMatch Repair
PCA: Principal Component Analysis
MTB: Molecular Tumor Board
RNAseq: RNA sequencing
