# Supplementary material for: From the identification of actionable molecular targets to the generation of faithful neuroblastoma patient-derived preclinical models
Source: J Transl Med. 2024 Feb 13;22:151. doi: 10.1186/s12967-024-04954-w (PMC10863144; doi:10.1186/s12967-024-04954-w)
Supplement: Supplementary file 1 — Additional file 1: Figure S1. Descriptive statistics of sequencing and mapping results from 50 samples A Box plot reporting the number of sequencing reads used for the alignment. B The percentage of bases with quality score above 30 (Q30). C The percentage of mapped reads (mapping rate). D The percentage of duplicated reads. E Box plot reporting the average sequencing depth on target obtained after the alignment and duplicates removal. F Box plot reporting the average coverage of target regions. G The percentage of target regions covered with at least 10 reads. H The percentage of target regions covered with at least 50 reads. The data values in panels A and E are shown by sample type and by sequencing panel. The data values in panels B, C, D, F, G and H are shown by sequencing panel type. Pz relapsed tumor. P1 first PDX/CPDX generation, P2 second PDX/CPDX generation. P2-3D 3D model derived from the second PDX/CPDX generation. Figure S2. Read counts and alignment statistics of RNAseq analysis. The figure summarizes the read counts and alignment statistics of each sample. A For each sample, we obtained from 10 to 20 million sequencing reads on average except for the sample Pz#C with more than 30 million reads. B On average, 80% of total reads were uniquely mapped to the genome, and 16% were mapped to multiple loci. Figure S3. Comparison of variant allele frequency among patient’s tumors and PDX/CPDX generations. Box plot showing the distribution of the variant allele frequency. Pz patient’s tumor, Pl first PDX/CPDX generation, P2 second PDX/CPDX generation. The values of each patient are represented as dots on each boxplot, and they are distinguishable based on the color code provided on the right side of the figure. Each color corresponds to a specific group or category of patients, allowing for easy identification and comparison of data across different groups. Figure S4. 3D models. The figure shows representative pictures of 3D/patient-derived tumor-spheres, devel [file 12967_2024_4954_MOESM1_ESM.docx]

**Additional file 1: Figure S1** | **Descriptive statistics of sequencing and mapping results from 50 samples**

**A** | Box plot reporting the number of sequencing reads used for the alignment. **B** | The percentage of bases with quality score above 30 (Q30). **C** | The percentage of mapped reads (mapping rate). **D** | The percentage of duplicated reads. **E** | Box plot reporting the average sequencing depth on target obtained after the alignment and duplicates removal. **F** | Box plot reporting the average coverage of target regions. **G** | The percentage of target regions covered with at least 10 reads. **H** | The percentage of target regions covered with at least 50 reads. The data values in panels **A** and **E** are shown by sample type and by sequencing panel. The data values in panels **B**, **C**, **D**, **F**, **G** and **H** are shown by sequencing panel type. Pz: relapsed tumor. Pl: first PDX/CPDX generation. P2: second PDX/CPDX generation. P2-3D: 3D model derived from the second PDX/CPDX generation.

**Additional file 1: Figure S2 | Read counts and alignment statistics of RNAseq analysis.**

The figure summarizes the read counts and alignment statistics of each sample. **A |** For each sample, we obtained from 10 to 20 million sequencing reads on average except for the sample Pz#C with more than 30 million reads. **B |** On average, 80% of total reads were uniquely mapped to the genome, and 16% were mapped to multiple loci.

**Additional file 1: Figure S3 | Comparison of variant allele frequency among patient’s tumors and PDX/CPDX generations.** Box plot showing the distribution of the variant allele frequency. Pz: patient’s tumor. Pl: first PDX/CPDX generation. P2: second PDX/CPDX generation. The values of each patient are represented as dots on each boxplot, and they are distinguishable based on the color code provided on the right side of the figure. Each color corresponds to a specific group or category of patients, allowing for easy identification and comparison of data across different groups.

**Additional file 1: Figure S4** | **3D models.|**

The figure shows representative pictures of 3D/patient-derived tumor-spheres, developed by using either tumor cells derived from first (P1) and second (P2) generation of PDX and CPDX or tumor cells directly derived from patient (Pz#C). Bar: 100 μm.

**Additional file 1: Figure S5** | **Comparison of genomic variations between patient’s tumors, PDX/CPDX generations and 3D models.**

The figure shows the results of analysis based on the variant allele frequencies of all the detected variants in the group of four tumors having PDX/CPDX samples up to second generation and 3D model production. Pz: **patient’s tumors**. Pl: first PDX/CPDX generation. P2: second PDX/CPDX generation. P2-3D: 3D model grown from the second murine xenograft generation. **A** | Clustered heatmap of the correlation coefficients. **B** | Hierarchical clustering using Euclidian distance and Ward’s method. **C** |Principal Component Analysis and Scree Plot (bottom right). **D** | Box plot showing the distribution of the variant allele frequency. The values of each patient are represented as dots on each boxplot, and they are distinguishable based on the color code provided on the right side of the figure. Each color corresponds to a specific group or category of patients, allowing for easy identification and comparison of data across different groups

**Additional file 1: Figure S6** | **Tracking of potential therapeutically targetable somatic alterations in PDX or CPDX generations and 3D models.**

**A** | The figure shows the allele frequency of targetable somatic variants detected in patient’s tumors and tracked in murine xenografts generations and 3D models derived from the second tumor generation. **B** | The figure shows the number of copies of potentially targetable somatic copy number variants detected in in patient’s tumors and tracked in murine xenografts generations and 3D models derived from the second tumor generation. Pz: primary tumor. Pl: first PDX/CPDX generation. P2: second PDX/CPDX generation.
